# Inferring lifestyle for Aves and Theropoda: A model based on curvatures of extant avian ungual bones

**DOI:** 10.1371/journal.pone.0211173

**Published:** 2020-02-05

**Authors:** Savannah Elizabeth Cobb, William I. Sellers

**Affiliations:** School of Earth and Environmental Sciences, The University of Manchester, Manchester, United Kingdom; Monash University, AUSTRALIA

## Abstract

Claws are involved in a number of behaviours including locomotion and prey capture, and as a result animals evolve claw morphologies that enable these functions. Past authors have found geometry of the keratinous sheath of the claw to correlate with mode of life for extant birds and squamates; this relationship has frequently been cited to infer lifestyles for Mesozoic theropods including *Archaeopteryx*. However, many fossil claws lack keratinous sheaths and thus cannot be analysed using current methods. As the ungual phalanx within the claw is more commonly preserved in the fossil record, geometry of this bone may provide a more useful metric for paleontological analysis. In this study, ungual bones of 108 birds and 5 squamates were imaged using X-ray techniques and a relationship was found between curvatures of the ungual bone within the claw of pedal digit III and four modes of life; ground-dwelling, perching, predatory, and scansorial; using linear discriminant analysis with weighted accuracy equal to 0.79. Our model predicts arboreal lifestyles for *Archaeopteryx* and *Microraptor* and a predatory ecology for *Confuciusornis*. These findings demonstrate the utility of our model in answering questions of palaeoecology, the theropod-bird transition, and the evolution of avian flight. Though the metric exhibits a strong correlation with lifestyle, morphospaces for PD-III curvatures overlap and so this metric should be considered alongside additional evidence.

## Introduction

The amniote claw is utilised in multiple functions related to ecology and lifestyle. Claws bear an animal’s weight during locomotion, are utilised in prey capture, and more. These tasks exert selective pressure on the claw and so claws are expected to evolve morphologies that enable performance of essential functions whilst minimising stress/strain during locomotion [1,2]. The evolutionary reduction of bending stresses during terrestrial locomotion is the proposed cause of the relatively flat claws observed for ground-dwelling taxa compared to pedal claws belonging to arboreal and/or predatory taxa, which tend to possess more curved claws for enabling grip [3–12].The relationship between claw morphology and lifestyle has frequently been utilised to infer lifestyle for fossil taxa.

Curvature of extant claws as quantified using “claw angle”, or the angle found for the arc of a circle approximated using points on the claw [13], has been shown to correlate with lifestyle for a diverse group including avians, squamates, and mammals, though results vary and classification can be difficult for animals with intermediate behaviours and/or claw angles [3–8,11,12]. Though curvature and other aspects of external claw geometry (i.e. measures taken on sheaths, toe pads, and skin) are known to correlate with lifestyle and locomotor ability [3–14], internal structure of the claw has not been sufficiently studied to this purpose.

Amniote claws are comprised of a terminal phalanx encompassed by an external keratinous sheath [15]. For avian and reptilian claws, these terminal phalanges are known as ungual bones, and the external sheaths are comprised of rigid, insoluble β-keratins and ‘soft’ α-keratins. Using Extant Phylogenetic Bracketing (EPB) [16], we infer non-avian theropod dinosaurs and other fossil taxa within Theropoda and Aves would have possessed claws of similar composition with comparable material properties [17,18]. This presumed compositional similarity, relatively comparable biomechanics of locomotion (e.g. bipedalism), and the strong trend toward convergence driven by major habits such as predation lead us to assume that fossil taxa on the avian lineage evolved similar claw morphologies to extant birds in response to similar ecological and functional pressures.

Ideally, one would compare extant and fossil claw sheaths, as the sheath interacts directly with the environment and is likely more biomechanically relevant. Theropod claw sheaths are rare but not unknown from the fossil record [19–26], and for these exceptional specimens analyses of the sheath are very useful. However, for most fossil specimens, the claw sheath is either broken or entirely absent leaving only the ungual bone, and fossilised toe pads or skin are even rarer [27–38].

As a result, measurements of fossil claw angle are often based on reconstructions [4] or taken directly on ungual bones [5,39]. Past attempts to find a relationship between curvature of the keratinous sheath and the ungual bone for reconstruction purposes have yielded variable results [4,8,40–42], and so at present reconstructed claw angles are based either on supposition or on a relationship for which no consensus has been reached. Analysing ungual bone curvatures with a model based on sheath curvatures is also likely to cause errors of misclassification, as ungual bones must be relatively short and flat to fit within the sheath. We suspect previous predictions of terrestrial lifestyle for fossil taxa may result from low angles of curvature inherent to ungual bones rather than true ecological signal [5,39].

One way to overcome limitations of past studies is to study the ungual bone so that fossil claws not preserving soft tissues can be directly compared to extant claws. If the ungual bone proves a useful metric, this will enable analysis for a greatly-expanded dataset of fossil material. Additionally, soft tissue reconstruction would be made unnecessary in such study if ungual bones alone provide an accurate, precise proxy for lifestyle. However, very little is known about avian or squamate ungual bone morphology.

This study investigates the relationship between dorsal and ventral curvatures of ungual bones and behavioural categories terrestrial, perching, predatory, and scansorial for a diverse group of extant avians and squamates. The results are then utilised to infer lifestyle for a sample of fossil paravians and avians.

## Materials and methods

We examined curvatures for ungual bones and sheaths belonging to 95 species of bird representing 25 orders and 5 species of squamate. As this study seeks to infer modes of life for fossil taxa on the avian lineage, the final predictive model is based on bird claws. A small group of behaviourally diverse squamates have also been tested to investigate if determined trends are universal or constrained by clade. Crocodilians have been excluded because modern taxa lack the behavioural diversity this study seeks to analyse.

Most analysed extant specimens were imaged on-site at the Manchester Museum (MANCH), the World Museum in Liverpool (NML), and the National Museum of Scotland in Edinburgh (NMS). Extant specimens were also acquired from Elaine Potter of the Predatory Bird Monitoring Scheme (PBMS) at the Lancaster Centre for Ecology & Hydrology (CEH) and game keepers. Full specifications of extant specimens are listed in S1 Table.

Photographs of PD-III claws from fossil paravians and avians were acquired from published sources (S2 Table). The analysed fossil specimens belong to institutions including the Geological Museum of Peking University (PKUP), Liaoning Palaeontological Museum (LPM), Beijing Museum of Natural History (BMNHC), Museum für Naturkunde Berlin (MB, MfN), Transylvanian Museum Society in Cluj (EME), Institute of Paleobiology in Warsaw (ZPAL), Paleontological Center at Bohai University (HG), Institute of Vertebrate Paleontology and Paleoanthropology (IVPP) of Chinese Academy of Sciences, Shandong Tianyu Museum of Natural History (STM), Yizhou Fossil & Geology Park (YFGP), Institute of Paleontology and Geology (MPC) at Mongolian Academy of Sciences, Chinese Academy of Geological Sciences (CAGS), Henan Geological Museum (HGM) in Zhengzhou, China, Dalian Natural History Museum (DNHM) in Liaoning, China, Natural History Museum of Utah (UMNH), and the Jinzhou Paleontological Museum (JPM).

Each specimen was placed into one of four behavioural categories; terrestrial, perching, predatory, or scansorial; based on the literature [43–55]. Behavioural complexity presents an issue, as few animals exhibit behaviours of just one defined group (for example, galliforms also roost in trees). Species have been selected to best represent each group as determined by percent life spent engaging in the assigned behaviour and possession of adaptations. Specimens have been selected such that each behavioural category includes three or more orders and some orders are represented within multiple behavioural categories. All extant specimens measured are adults to constrain potential influences of ontogenetic and/or behavioural changes during life known for some taxa [56].

Claw angles from predatory taxa have been reported to overlap significantly with other groups [8], and so birds that utilise talons to dispatch and/or capture prey have been included with the intent of reducing Type II error (e.g. misclassification of predatory/scansorial claws with similar curvatures). The predatory sample group is comprised of extant raptors (Accipitriformes, Strigiformes, Falconiformes). Though claw taper is purported to distinguish predatory from non-predatory birds [57], this has not been sufficiently demonstrated for the ungual bone. Finding a distinctive morphospace based on predatory claw curvatures is of interest for various enquiries and will expand the predictive capacity of the model to include ecological as well as locomotor groupings.

Claws were radiographed in lateral view using the Nomad Pro Radiography Unit and processed in the SIDEXIS software (https://www.dentsplysirona.com/en). For small claws less than roughly 10 mm long, sheath data were supplemented with photographs when necessary, and multiple images were taken from a fixed orientation in space at different exposures and then layered to create a weighted average in Helicon Focus 7 to improve edge unsharpness at the claw tip. This technique is known as High Dynamic Range (HDR) Imaging. [58].

The Nomad Pro Radiography Unit has a lithium polymer battery that charges at 110/220 V and operates at 22.2 V and 1.25 Ahr. The anode voltage is 60 kV true DC and the anode current is 2.5 mA. We utilised the Xios XG Supreme digital sensor, which has a theoretical resolution of 33 Lp/mm (Line pairs per millimetre) and 15 μm pixel size, a measured resolution of 28 Lp/mm, an active sensor area of 25.6 x 36 mm, and external dimensions of 31.2 x 43.9 x 6.3 mm (https://www.dentsplysirona.com/en).Maximum dose of radiation to the user registered between 0.0117 mGy and 0.0310 mGy at the palm depending on how the device is positioned [59]. All measured dosages are well below permissible limits according to the Ionising Radiation Regulations 2017 [60].

This device enabled the rapid, inexpensive, on-site acquisition of a large data set and is a practical alternative to CT scanning. However, more refined techniques such as geometric morphometric (GM) analysis may not be feasible based on pixel count and so linear measurements have been taken and analysed.

Fossil specimens include paravians, avialans, and avians and were selected based on condition, image resolution, and phylogenetic closeness to extant birds. The selected fossil claws show no significant breakage or distortion, and so we assume measured values represent true claw angles of the animal during life. Slight reconstruction was necessary for all fossil sheaths and for ungual bones belonging to two troodontid specimens *Talos* and MPC-D 100/140.

Claw length was limited to a maximum of 44 mm to fit on the active sensor area and a minimum of 7 mm because fine details could not be resolved for very small claws. As body mass and claw radius correlate [8], body masses for the sample taxa are limited from 36g to 1930g to constrain claw size.

Body masses were determined from the CRC Handbook of Avian Body Masses [61] and may differ slightly from true body masses of individuals as this information was often unavailable for the sample specimens. When it was not possible to sex the specimens, body mass was calculated as the average of male and female body mass. We regressed body mass against claw angle for each behavioural category to determine effects of scaling.

Claw curvature among digits can vary greatly [40] and so we have constrained digit of study to pedal digit III (PD-III) after the fashion of past studies. Recent studies indicate analysis of multiple digits is more informative than analysis of a single digit [40,62]; the choice of PD-III was a pragmatic necessity given the time available. Digit III is the central and longest digit for many birds, squamates, and non-avialan Mesozoic theropods and represents the first and last point of contact with the substrate during terrestrial locomotion. As it is functionally significant, claw morphology of digit III is expected to be well-adapted for locomotion and other functions [4–6,14,63].

Due to phylogenetic conflict within Aves and the difficulties in placing fossil taxa phylogenetic corrective methods were not used. Two recent studies resolved conflicting topologies for Aves [64,65], and phylogenies for fossil taxa are less stable.

### Geometric measurements

**Fig 1:**
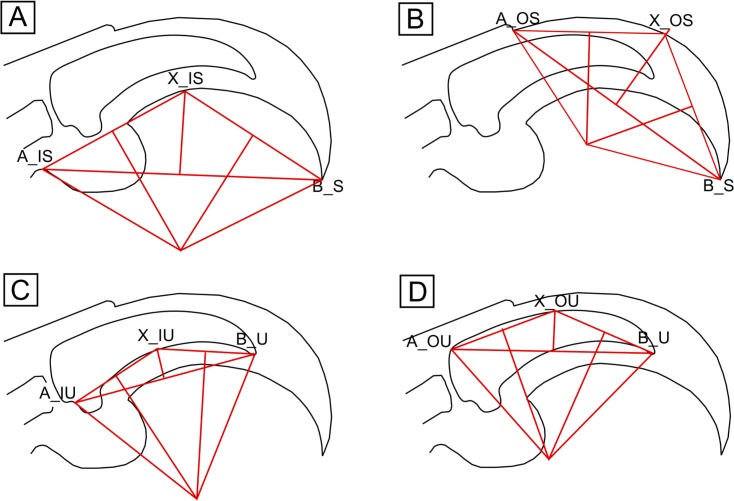
Methods of determining claw angle. Claw drawn in Inkscape. Measurements performed in DinoLino.exe. Landmark A is located at the proximal termination of the dorsal (A_OS) surface of the sheath or ungual bone (A_OU), or the fleshy (A_IS) or bony (A_IU) base of the flexor tubercle. Landmark B is located at the tip of the ungual bone (B_U) or sheath (B_S). Landmark X is drawn at the intersection of $\bar{AB}’s$ bisector with the curved surface being measured. (A) Feduccia’s [3] method of quantifying inner curvature of the claw sheath, here denoted IS. (B) Pike and Maitland’s [8] method of quantifying outer curvature of the claw sheath, here denoted OS. (C) A modification of Feduccia’s method to measure inner curvature of the ungual bone, here denoted IU. (D) A modification of Pike and Maitland’s method to measure outer curvature of the ungual bone, here denoted OU.

**Fig 2:**
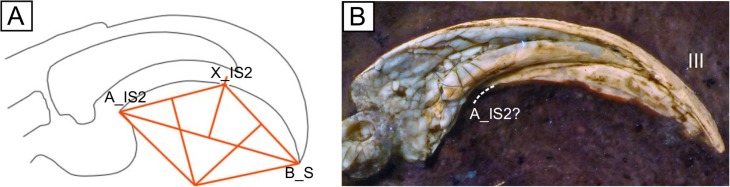
Alternative measure utilised for fossil claws and example *Archaeopteryx* claw. Claw drawn in Inkscape; measurements performed in DinoLino.exe. Landmark A_IS2 is located at the proximal termination of the ventral surface of the sheath. (A) A method of measuring claw angle after Fowler and colleagues [40], here denoted IS2. (B) Right pedal claw from the 12^th^ Archaeopteryx specimen [21]. Slight reconstruction was necessary for approximating landmarks.

Angles of curvature for dorsal or ventral surfaces of each claw were calculated using three landmark points A, B, and X on the claw, which vary in position depending on metric (Figs 1 and 2). Ventral and dorsal curvatures of the ungual bones (IU, OU) and claw sheaths (IS, IS2, OS) were measured according to the methods outlined in Feduccia [3], Pike and Maitland [8], and Fowler and colleagues [40]. The centre of a circle O is drawn at the intersection of perpendicular bisectors to $\bar{AX}$ and $\bar{BX}$, and claw angle is taken as ∠AOB [66]. Custom software was created in Microsoft Visual Studio using C++ for taking measurements with improved speed and precision as compared to other available programs (https://github.com/johnwelter/Dino-Lino).

Two measures (IU, OU) were taken on fossilised ungual bones. For fossil claws in possession of claw sheaths, ventral curvature of the sheath was measured using a method excluding the toe pad (Fig 2) that has also been shown to correlate with modes of life [13]. This measurements shifts landmark A distally to the start of the ventral sheath but retains landmark B at the tip of the claw sheath as this can often be directly measured on the fossil claws (Fig 2). Toe pads are not known for any of the measured fossil claws, and so using this measurement as opposed to IS minimises the amount of reconstruction necessary (Fig 2).

Including measures of both inner and outer curvature provides a proxy for claw height and degree of taper (e.g. a claw with outer curvature much lower than inner curvature will have a tall base and high taper). However, the variable size and position of the flexor tubercle may lead to inconsistency in measurements of inner curvature. We recommend future workers utilise outer curvature and a measure of height at the base and/or midpoint.

### Statistical analysis

Statistical analyses and graphical summaries were performed in R [67]. Body mass has previously been shown to have a complex relationship with claw morphology [8], and so we tested for a linear relationship between body mass and claw angle by group for each measure taken on avian ungual bones and claw sheaths. Simple linear regression was performed by group on body mass and claw angles for the dataset of extant avians using the smatr package [68]. To normalise the data, body mass was log-transformed and claw angle was transformed by cosine. As variances differ among behavioural groups (heteroscedasticity), pairwise and non-parametric median and permutational tests were utilised to determine if median claw angles and/or centroids of combined measures of claw angle differ by group. The statistically significant p-value was defined as the standard 0.05.

Measures were submitted to linear discriminant analyses using the caret package in R [69]. Predictive models were created using four subsets of the avian data: ungual bone measurements (IU, OU), claw sheath measurements (IS, OS), sheath and bone measurements (IU, OU, IS, OS), and sheath and bone measurements with the modified measure IS2. Predictive success of the models was tested for extant birds using bootstrap resampling with 2000 iterations using the train function of the caret package [69]. Predictions were generated for fossil taxa using the determined models.

## Results

### Relationship to body mass

**Fig 3:**
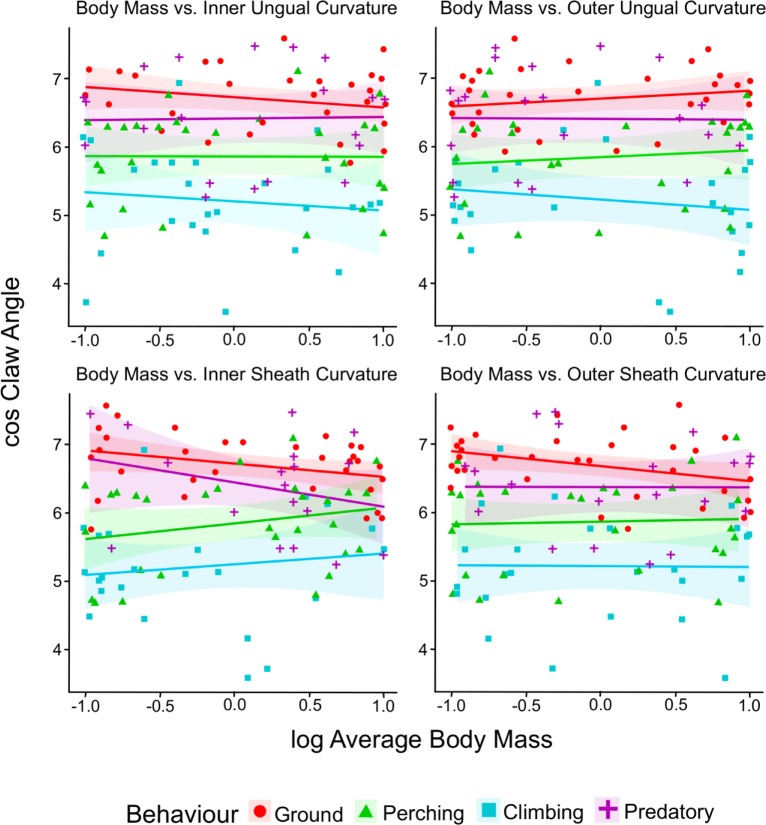
Regression plots for claw angle against body mass grouped by behavioural category. The shaded area represents 95% confidence limits of the regressions. Only one of the regressions is statistically significant.

Fig 3 clearly shows that very little of the variation in claw angle can be explained by body mass although there is a weak association with outer curvature of the keratinous sheath. Ungual bones do not show a statistically significant relationship with body mass for the sample taxa. There is a negative relationship between dorsal claw angle of the sheath (OS) and body mass for terrestrial taxa (p = 0.04641). However, the amount of data explained by the linear best fit line between OS angles and body masses of terrestrial taxa was low (R^2^ = 0.1219), suggesting other factors are influencing this relationship.

### Claw geometry and behavioural category

**Fig 4:**
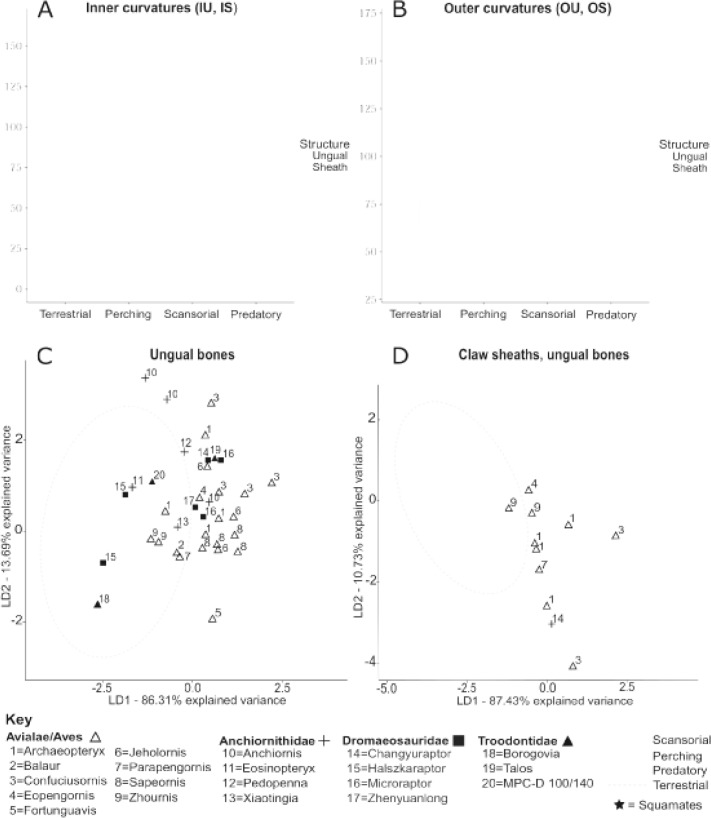
Curvatures of ungual bones and claw sheaths, digit III, for all extant and fossil taxa. Boxplots for inner (A) and outer (B) claw curvatures distinguished by behavioural category for all extant taxa. Shaded boxes depict interquartile range (IQR). Whiskers depict distance between IQR and points up to 1.5 distances from the IQR. Outliers are represented with circles or stars dependent on taxonomic group and are greater than 1.5 distances from the IQR. Morphospaces based on LD1 and LD2 generated by an LDA of combined ungual bone measures (C) and combined sheath and bone measures (D) for extant birds, overlain with data for fossil claws. Ellipses were drawn with 95% confidence from the centroid for each group.

The relationship found for ungual bones is similar to that found for claw sheaths: lower angles of curvature correlate with terrestrial lifestyles, intermediate claw angles correlate with perching and predatory lifestyles, and higher claw angles correlate with scansorial lifestyles (Fig 4). Compared to claw sheaths, ungual bone curvatures had relatively constrained ranges and possessed lower values of claw angle. Perching, ground-dwelling, and climbing taxa had roughly equivalently sized ranges of ungual bone curvatures. Predatory taxa had the smallest ranges of claw angle but, notably, were represented by the fewest sample specimens. Median values of inner curvature were much lower than those of outer curvature in all but the ‘predatory’ group.

**Fig 5:**
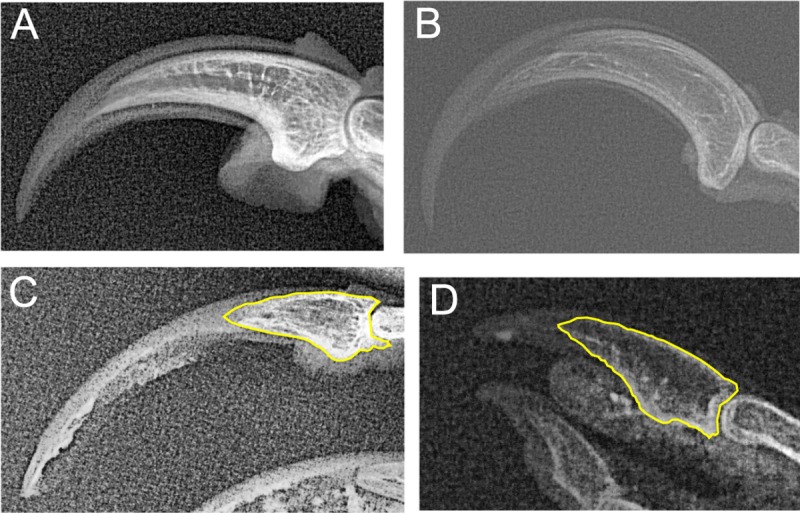
Radiographs of pedal digit III claws exhibiting significant morphological disparity. Ungual bones outlined in yellow have inner curvatures measured at zero. (A) Right claw of kakapo (*Strigops habroptila*). Specimen 1882.41.3, National Museum of Scotland (B) Left claw of ivory-billed woodpecker (*Campephilus principalis*). Unregistered skin, National Museum of Scotland. (C) Right claw of willow ptarmigan (*Lagopus lagopus*). Skin specimen 1984.2.197, Liverpool World Museum. (D) Left claw of gull (*Larus canus*). Skin specimen 1384, National Museum of Scotland.

All squamate claws plotted as outliers and have been excluded from further analyses for their likely incomparability with extant avians and fossil theropods. Ranges for measures taken on claw sheaths, particularly inner sheath curvatures, showed more outliers than those measured for ungual bones, suggesting high variability of ungual soft tissues relative to bone. Of the sampled avian ungual bones, extreme values are apparent in the ‘terrestrial’ category showing inner curvatures measured at zero: *Numenius arquata* (Eurasian curlew), *Larus canus* (common gull), and *Lagopus lagopus* (willow ptarmigan). Gulls and curlews have aquatic habits and webbed feet, which may have influenced the evolution of very flat ungual bones, and the willow ptarmigan has a visibly unusual claw morphology (Fig 5). Avian claws display a wide range of morphologies and so we believe these nearly flat ungual bones, though plotted here as extreme values, represent normal diversity in the population. The above-mentioned avian taxa have thus been retained in the analysis.

For the model based solely on two ungual bone measurements (Fig 4), two axes LD1 and LD2 are generated that account for all variation. For the model utilising four measurements (Fig 4), LD1 and LD2 account for 97% of the variation, and so LD3 is not likely to significantly impact separation. LD1 represents greater or lesser values of claw angle for any given metric, accounts for greater than 85% of variation, and separates terrestrial taxa from predatory/perching taxa from scansorial taxa. Claws of perching birds tended to have lower LD2 values compared to claws of predatory birds.

Despite differing centroids (Table 2), there is overlap between ranges for each group. Morphospaces created based on measures of avian ungual bones overlap between all categories except ‘terrestrial’ and ‘scansorial’. Those based on all claw measures have similar degrees of overlap but manage to also separate ‘terrestrial’ and ‘predatory’ groups.

**Table 1:** Summary from a simple linear regression on cos(claw angle) against log(average body mass).

Metric	Behaviour	Slope	Intercept	p-value	R^2
**Inner Ungual Curvature (IU)**	**Terrestrial**	-0.3324	2.4224	0.2277	0.04658
	**Perching**	-0.00934	0.021829	0.9658	6.94E-05
	**Predatory**	0.02547	-0.11135	0.9117	0.00063
	**Scansorial**	-0.0817	0.3474	0.6211	0.0108
**Outer Ungual Curvature (OU)**	**Terrestrial**	0.3062	-2.153	0.2983	0.03484
	**Perching**	0.1257	-0.7192	0.58	0.01149
	**Predatory**	-0.01407	-0.13774	0.95	0.000202
	**Scansorial**	-0.1542	0.8701	0.4757	0.02235
**Inner Sheath Curvature (IS)**	**Terrestrial**	-0.5287	3.6631	0.07161	0.1009
	**Perching**	0.2348	-1.3762	0.2271	0.05354
	**Predatory**	-0.2106	1.5779	0.2314	0.07446
	**Scansorial**	0.1219	-0.8576	0.5081	0.01927
**Outer Sheath Curvature (OS)**	**Terrestrial**	-0.5597	3.6567	0.04641	0.1219
	**Perching**	0.04641	-0.4269	0.8295	0.001748
	**Predatory**	-0.00565	0.112745	0.9793	0.00069
	**Scansorial**	-0.00768	0.009928	0.9681	7.11E-05

**Table 2:** Results of non-parametric tests performed on subsets of the avian data. P-values represent Bonferroni-corrected values.

	Wilcoxon Rank Sum			PERMANOVA		
	**Inner ungual curvature (IU)**			**All ungual curvatures (IU, OU)**		
	**Climbing**	**Terrestrial**	**Perching**	**Climbing**	**Terrestrial**	**Perching**
**Terrestrial**	1.07E-09	-	-	0.0006	-	-
**Perching**	1.30E-10	4.01E-06	-	0.0006	0.0006	-
**Predatory**	7.13E-03	1.25E-07	4.29E-4	0.0006	0.0006	0.0036
	**Outer ungual curvature (OU)**			**All measures (IU, OU, IS2, OS)**		
**Terrestrial**	1.07E-09	-	-	0.0006	-	-
**Perching**	5.60E-08	2.84E-06	-	0.0006	0.0006	-
**Predatory**	1.36E-06	1.48E0-05	1	0.0006	0.0006	0.0736

Centroids of inner and outer curvatures for ungual bones differed between all behavioural categories except the ‘perching’ and ‘predatory’ categories (Table 2). This is not unexpected as box plots shows inner and outer curvature values of the ‘predatory’ group completely overlap with ranges found for the ‘perching’ group (Fig 4). Pairing inner and outer curvatures of the ungual bone makes it possible to distinguish between perching and predatory claws; centroids based on these values differed significantly between all groups. Interestingly, including soft tissue measures in the multivariate analysis worsened separation among groups. Centroids based on four measures of sheath and ungual curvatures differed significantly between all groups except for perching and predatory taxa, the comparison of which yielded a p-value equal to 0.0736 after Bonferroni correction.

Predictive models based on ungual bone curvatures had greater success at classing extant bird claws (total accuracy = 0.7865) relative to models based on similar measures taken on keratinous sheaths (total accuracy = 0.7273). Both predictive models based on bone and sheath measurements had relatively greater success than models based on only two measures (Table 3). Utilising the modified sheath measure IS2 as opposed to IS slightly reduced predictive success (total accuracy = 0.8103) but did not greatly alter results relative to the model utilising IU, OU, IS, and OS (total accuracy = 0.8190).

**Table 3:** Predictive success of the models based on extant bird claws. Accuracy for each behavioural category and weighted total accuracy are listed.

Included Measures	Accuracy: Terrestrial	Accuracy: Perching	Accuracy: Scansorial	Accuracy: Predatory	Accuracy: Total
**Unguals (IU, OU)**	0.8182	0.658	0.813	0.8906	0.7865
**Unguals, sheaths (IU, OU, IS, OS)**	0.8078	0.7948	0.8276	0.8594	0.819
**Unguals, sheaths (IU, OU, IS2, OS)**	0.8279	0.7138	0.8347	0.8854	0.8103
**Sheaths (IS, OS)**	0.7915	0.6506	0.6667	0.8083	0.7273

Perching birds are consistently classed with the lowest accuracies ranging from 0.6506 to 0.7948 predictive success, and predatory taxa are classed with the highest accuracies ranging from 0.8083 to 0.8906 predictive success dependent on the dataset. Accuracies when classing terrestrial and scansorial taxa exceed 80% and were of similar values for the models based on all subsets except that based on sheaths, for which accuracy when classing terrestrial taxa (0.7915) was greater than accuracy when classing scansorial taxa (0.6667). Variance-covariance matrices for the predictive models are provided in Table 4.

**Table 4:** Variance-covariance loadings for each variable included in the analysis. (A) Variance matrix based on LDA of all measures. (B) Variance matrix based on LDA of ungual bones.

**Model based on all claw measures (IU, OU, IS2, OS)**			
**Measure**	**LD1**	**LD2**	**LD3**
**Inner ungual curvature (IU)**	0.039494	0.085473	-0.01741
**Outer ungual curvature (OU)**	0.013435	-0.11955	-0.07304
**Inner sheath curvature (IS2)**	0.022785	-0.00956	0.025289
**Outer sheath curvature (OS)**	0.001744	0.031581	0.044619
**Model based on ungual bone measures (IU OU)**			
**Measure**	**LD1**	**LD2**	**NA**
**Inner ungual curvature (IU)**	0.041889	0.082856	NA
**Outer ungual curvature (OU)**	0.033374	-0.09416	NA

### Comparison with fossil taxa

For both LDA plots based either on ungual bone data or all claw measures, the majority of fossil taxa plot in the predatory/perching morphospaces (Fig 4). Dromaeosaurid and anchiornithid ungual bones plot in predatory, perching, and terrestrial morphospaces, and avialan ungual bones plot in predatory, perching, and scansorial morphospaces. For both LDA plots based either on ungual bone data or all claw measures, the majority of fossil claws plot within the overlapping regions of the 95% confidence ellipses, but some taxa plot in regions distinct to a particular morphospace. These include fossil dromaeosaurid *Halszkaraptor* and anchiornithid *Eosinopteryx*, which all plot as terrestrial, and dromaeosaurid *Zhenyuanlong*, which plots as perching. None of the fossil claws plot as distinctly scansorial though some avialan claws including *Sapeornis*, *Confuciusornis*, and *Fortunguavis* plot in the shared spaces between predatory-scansorial and perching-scansorial taxa (Fig 4).

In the LDA plot based on ungual bone data, fossil claws representing two *Anchiornis* and one *Confuciusornis* specimen represent outliers (Fig 4C). One *Archaeopteryx* claw lies just outside the ‘predatory’ morphospace, but when measures of the sheath are included in the morphospace analysis, this data point shifts such that it is encompassed by the ‘predatory’ morphospace. In the LDA plot based on all claw data (Fig 4D), two *Confuciusornis* claws including the outlying specimen from Fig 4C plot as outliers. It is unknown whether the outlying *Anchiornis* claws from Fig 4C would also be outliers in Fig 4D when considering all claw data as sheath data were unavailable for the measured specimens.

Claws belonging to *Archaeopteryx*, *Confuciusornis*, and *Microraptor* had a wide spread. *Archaeopteryx* specimens plotted within all morphospaces, and right and left PD-III claws of the same specimen plotted quite far apart from each other. This was also the case for *Microraptor*, for which two claws belonging to a single specimen plotted in different morphospaces (predatory and perching) and received different classifications accordingly (Table 5). Though all were classed as predatory (Table 5), the four *Confuciusornis* claws were recovered in the overlaps between predatory, perching, and scansorial morphospaces and, in some instances, plotted outside of extant morphospaces altogether (Fig 4).

**Table 5:** Posterior probabilities for fossil claws ecological grouping. Predictions were generated for fossil taxa by two predictive models: Model 1 based on ungual bone curvatures, and for claws in possession of the keratinous sheath, Model 2 based on ungual bone and sheath curvatures. GRND = Ground-dwelling, PRCH = Perching, CLMB = Climbing, PRED = Predatory. Highlighted cells indicate classification for the claw. Yellow = classification by Model 1 alone (no sheath data), green = classification for which Models 1 and 2 are in agreement, red = classification for which Models 1 and 2 are in disagreement.

CLAW DETAILS			MODEL 1 (IU + OU)				MODEL 2 (IU + OU + IS2 + OS)			
Binomial	Specimen	Pes	GRND	PRCH	CLMB	PRED	GRND	PRCH	CLMB	PRED
*Anchiornis huxleyi*	PKUP V1068	R	0.061	0.092	0.001	0.846	NA	NA	NA	NA
*Anchiornis huxleyi*	LPM-B00169	R	0.026	0.424	0.078	0.472	NA	NA	NA	NA
*Anchiornis huxleyi*	BMNHC PH823	R	0.161	0.075	0.000	0.765	NA	NA	NA	NA
*Archaeopteryx sp*.	12th specimen	R	0.044	0.615	0.116	0.225	0.274	0.509	0.026	0.191
*Archaeopteryx sp*.	12th specimen	L	0.009	0.114	0.010	0.867	0.010	0.102	0.003	0.885
*Archaeopteryx sp*.	Berlin specimen	L	0.016	0.447	0.172	0.366	0.005	0.450	0.063	0.482
*Archaeopteryx sp*.	Berlin specimen	R	0.325	0.524	0.007	0.144	0.066	0.716	0.006	0.212
*Balaur bondoc*	EME PV.313	L	0.218	0.687	0.025	0.070	NA	NA	NA	NA
*Borogovia gracilicrus*	ZPAL MgD-I/174	R	0.952	0.048	0.000	0.000	NA	NA	NA	NA
*Changyuraptor yangi*	HG B016	L	0.014	0.199	0.024	0.764	0.005	0.125	0.002	0.868
*Confuciusornis sanctus*	IVPP V13156	L	0.011	0.311	0.098	0.580	0.000	0.205	0.046	0.750
*Confuciusornis sanctus*	MB.Av.1158	R	0.003	0.040	0.004	0.954	0.000	0.020	0.000	0.980
*Confuciusornis sanctus*	IVPP V 14412 A	R	0.002	0.168	0.271	0.560	NA	NA	NA	NA
*Confuciusornis sanctus*	IVPP V 13156	L	0.000	0.056	0.447	0.498	NA	NA	NA	NA
*Eopengornis sp*.	STM24-1	L	0.047	0.460	0.045	0.448	0.404	0.359	0.016	0.221
*Eosinopteryx brevipenna*	YFGP-T5197	R	0.747	0.201	0.000	0.052	NA	NA	NA	NA
*Fortunguavis xiaotaizicus*	IVPP V18631	?	0.027	0.609	0.345	0.020	0.041	0.256	0.004	0.699
*Halszkaraptor escuilliei*	MPC D-102/109	?	0.940	0.060	0.000	0.001	NA	NA	NA	NA
*Halszkaraptor escuilliei*	MPC D-102/109	?	0.817	0.155	0.000	0.028	NA	NA	NA	NA
*Jeholornis curvipes*	YFGP-yb2	L	0.005	0.310	0.296	0.389	NA	NA	NA	NA
*Jeholornis sp*.	STM2-37	R	0.017	0.234	0.029	0.720	NA	NA	NA	NA
*Jeholornis sp*.	STM2-37	L	0.020	0.552	0.257	0.171	NA	NA	NA	NA
*Microraptor zhaoianus*	CAGS 20-8-001	R	0.045	0.546	0.077	0.333	NA	NA	NA	NA
*Microraptor zhaoianus*	CAGS 20-8-001	L	0.005	0.151	0.041	0.803	NA	NA	NA	NA
*MPC-D 100/140*	MPC-D 100/140	L	0.456	0.362	0.002	0.181	NA	NA	NA	NA
*Parapengornis sp*.	IVPP V18632	L	0.192	0.708	0.031	0.069	NA	NA	NA	NA
*Pedopenna daohugouensis*	V12721	?	0.060	0.246	0.006	0.688	NA	NA	NA	NA
*Sapeornis chaoyangensis*	STM16-18	R	0.005	0.342	0.403	0.250	NA	NA	NA	NA
*Sapeornis chaoyangensis*	41HIII0405	L	0.021	0.551	0.231	0.197	NA	NA	NA	NA
*Sapeornis chaoyangensis*	41HIII0405	R	0.004	0.320	0.525	0.151	NA	NA	NA	NA
*Sapeornis chaoyangensis*	DNHM-D3078	L	0.056	0.678	0.115	0.151	NA	NA	NA	NA
*Talos sampsoni*	UMNH VP 19479	R	0.008	0.168	0.031	0.792	NA	NA	NA	NA
*Xiaotingia zhengi*	STM 27–2	L	0.202	0.633	0.019	0.147	NA	NA	NA	NA
*Zhenyuanlong suni*	JPM-0008	R	0.069	0.535	0.042	0.355	NA	NA	NA	NA
*Zhouornis hani*	BMNHC Ph 756	R	0.542	0.421	0.003	0.035	0.092	0.820	0.006	0.082
*Zhouornis hani*	BMNHC Ph 756	L	0.436	0.512	0.005	0.047	0.666	0.313	0.001	0.020

Classifications were often based on low to moderate posterior probabilities (Table 5). Most fossil ungual bones received predatory classifications (n = 15) followed by perching (n = 12) and ground-dwelling (n = 6) classifications. The same trend was apparent when sheath measures were included. Climbing classifications were observed only for two ungual bones from *Sapeornis*; the other two measured *Sapeornis* claws received perching classifications. There were multiple instances in which PD-III claws belonging to different feet of the same specimen received different classifications (*Archaeopteryx*, *Zhouornis*, *Sapeornis*, *Microraptor*).

The predictions based on all measures of claw curvature differ slightly from those based on ungual bone curvature. The right claw of the 12^th^ *Archaeopteryx* specimen shifts from perching to predatory, *Eopengornis martini* shifts from a perching classification to a terrestrial classification, *Parapengornis eurycaudatus* shifts from a perching classification to a predatory classification, and both Zhouornis claws shift with one receiving a ground-dwelling classification and the other receiving a perching classification. These shifts typically occurred when initial classifications were based on low posterior probabilities (Table 5).

## Discussion

### Utility of the model

Many previous studies have attempted to predict fossil lifestyles based on claw geometry, often utilising trends found for extant claw sheaths to classify fossil ungual bones. This study found a similar relationship between lifestyle and curvature for ungual bones as has been found for claw sheaths, but ungual bones possess relatively lower curvatures compared to sheaths (Fig 4) and thus cannot be directly compared to extant sheaths without risking misclassification. By determining the relationship between ungual bone geometry and certain lifestyles for a phylogenetically diverse sample of extant avians, this study overcomes limitations of past studies and enables direct comparison of fossil and extant material.

The claws of climbing squamates had higher outer curvatures of PD-III ungual bones compared to those of terrestrial squamates in the sample (Fig 4), which would suggest the results of this study represent a universal trend amongst tetrapods to some extent. However, our results indicate ungual bones of squamates have much lower claw angles than avian ungual bones and may be incomparable using these methods (Fig 4). This contradicts a study finding lizard claws to be generally congruent with bird claws by behavioural category [5], and so it is possible the effect stems from the very small sample size (n = 5) or perhaps ungual bones are affected more strongly by phylogenetic/biomechanical differences. Including a phylogenetic outgroup would increase confidence of assertions for fossil taxa, and so further work including more outgroup taxa may be useful.

No significant correlation was found between claw angle and body mass for ungual bones. Only claw sheaths of terrestrial taxa exhibited a statistically significant relationship with body mass (Table 1). The relationship found is poorer than reported for some previous studies [8,70] and roughly parallels findings of a recent comprehensive study [5]. These results suggest the correlations found in this study are relatively unaffected by body mass, and so the limited weight range of the extant sample taxa is not expected to have a significant impact on findings.

The predictive model based on ungual bone curvatures outperformed that based on claw sheath curvatures (Table 3), which suggests ungual bones provide a more accurate metric. This indicates reconstructing external sheath morphology is unnecessary for comparative analysis. However, predictions based on ungual bone curvatures alone were unstable and subject to change with the inclusion of sheath measures (Table 5). Models based on ungual bone and sheath curvatures yielded the most accurate predictions for extant taxa, and so when soft tissues are well-preserved in fossil claws we recommend following this approach.

Though median values for most behavioural categories could be separated based on one or more measures of claw curvature, there is significant overlap between the ellipses drawn for each behavioural category, particularly for that of claws belonging to perching birds. This results in frequent misclassifications of perching birds compared to other categories (Table 2). Predictions of lifestyle for fossil taxa based on claw curvature alone (or in fact any behavioural/ecological inference) should thus be considered alongside additional evidence to improve reliability of predictions [71,72].

The results show that reconstructing behaviour from D-III curvature is not particularly reliable (Table 5). There are a number of reasons why this may be the case. Behavioural complexity presents an issue for this and any study attempting to link morphology with mode of life [5,9]. Most animals utilise pedal claws for multiple functions to varying degrees, and so it is difficult to class any animal into a single behavioural category [4,5,8]. Many birds with perching or climbing adaptations also spend time foraging on the ground, for example, and all predatory birds measured for this study are also perching birds. For this reason, one could alternately interpret the ‘predatory’ morphospace as a ‘predatory-perching’ morphospace. Unfortunately, it was not feasible to include ground-dwelling taxa that utilise claws for prey capture/dispatching (e.g. secretary birds), and so we cannot test if there is a distinction between claw curvatures of terrestrial versus arboreal predators.

Previous studies have found conflicting results regarding which, if any, of the defined groups exhibits greater behavioural generalization with regard to claw shape [5,8]. The results of this study suggest claw sheaths may more greatly reflect behavioural generalization or specialization, while ungual bones appear to possess roughly equivalent spread by group (Fig 4). Ungual bone curvatures of the predatory sample taxa have the narrowest morphospace and seem to have similarly- curved claws. This may relate to biomechanics of piercing in prey capture and dispatching, or perhaps the predatory taxa could be interpreted as hyper-specialised perching birds. Alternatively, the narrow ‘predatory’ morphospace could be an artefact of the relatively small sample size for predatory claws.

The narrow predatory morphospace (Fig 4) and the high accuracy of the predictive model when classing predatory claws (0.89) (Table 3) indicate curvature is useful in distinguishing predatory talons. However, morphospaces overlap (Fig 4) and so there is still potential for Type II error. Including one or more additional measures of claw shape (e.g. base morphology, lateral compression) would likely improve separation between predatory and non-predatory claws.

In addition to the extant taxa measured, this study also measured 36 pedal claws from 28 specimens representing 19–20 genera of fossil dromaeosaurs, troodontids, anchiornithids, and avialans. Our results suggest scansorial habits for many of the measured fossil taxa with the majority grouping with perching birds and roughly two-thirds plotting outside the 95% confidence ellipse for terrestrial taxa (Fig 4). Many fossil taxa that grouped with perching birds lack an opposable hallux and would have been incapable of perching in the style of modern birds; also, the relative immobility of the theropod ankle joint would have likely prohibited movement along complex surfaces of the arboreal canopy [73]. However, these results could be interpreted as supporting some degree of arboreality (i.e. scansoriality) in Mesozoic theropods such as *Microraptor*, *Changyuraptor*, and *Pedopenna* that possess adaptations consistent with arboreal habits such as extensive hindlimb feathering and elongate forelimbs [24,74–76].

*Archaeopteryx* claws were classed as either perching or predatory, and three of four measured claws received low posterior probabilities (>0.05) for a terrestrial classification (Table 5). The results could be interpreted as suggestive of arboreal habits. Arboreal habits have been inferred based for *Archaeopteryx* based on mediolaterally compressed pedal claws similar to those of extant trunk-climbing birds [57,77], phalangeal proportions that indicate grasping ability of the pes [62], a hallux that was medially or perhaps even fully retroverted [57,78], and other features (e.g. swivel wrist joints, apparent gliding adaptations). [79,80]. Unfortunately, *Archaeopteryx* claws were well-scattered (Fig 4) and predictions varied dependent on claw (Table 5), and so we offer only tentative support for an arboreal interpretation of *Archaeopteryx* based on these results.

All *Anchiornis* claws received a predatory classification. Though anchiornithids possessed features consistent with a predatory lifestyle such as sharp teeth, *Anchiornis* claws plot well outside extant morphospaces (Fig 4) and as such are more useful as a cautionary example against analysing claws with unusual morphologies. The model utilises high LD2 values to distinguish the predatory morphospace, and so the model classes the *Anchiornis* claws as predatory because they possess very high values for LD2, despite data points falling outside extant morphospaces.

Of the sampled troodontids and dromaeosaurs, terrestriality/arboreality as measured along LD1 roughly correlates with phylogenetic status (Fig 4). It has been suggested based on pedal proportions, feather arrangement, and cranial capacities that over time, dromaeosaurids moved away from an ancestral cursorial state whilst troodontids became more adapted to cursoriality [62,79]. Our results could be interpreted as reflecting such a trend.

Our basal-most troodontid (*Talos*) has curved PD-III claws that plot with raptorial birds, the more derived MPC-D 100/140 plots closer to the terrestrial morphospace, and our most derived troodontid (*Borogovia*) has quite flat claws that recover within the terrestrial morphospace [81] (Fig 4). The predatory classification for *Talos* is based on a high posterior probability (0.792) and is supported by pathological modification of the left pes consistent with high predatory/defensive loadings [29]. The relatively flat claws of more derived troodontids may be adaptive to a specialist cursorial ecology, which is supported by postcranial features of these taxa (e.g. arctometatarsalian metatarsus, phalangeal proportions, elongate hindlimbs) [62].

The basal-most dromaeosaur measured (*Halszkaraptor*) plots with extant ground-dwellers, whereas multiple small, relatively derived dromaeosaurs fall within (*Microraptor*, *Changyuraptor*) or close to (*Zhenyuanlong*) the overlap between ‘predatory’ and ‘perching’ morphospaces (Fig 4) [81], All measured dromaesaurids possess features ascribed to a terrestrial/cursorial ecology (e.g. subarctometatarsalian metatarsus, hindlimb characters) [62,73] and so specialist arboreality seems unlikely. We hypothesize microraptorines *Changyuraptor* and *Microraptor* may have had scansorial habits based on their low posterior probabilities for terrestriality (>0.05) (Table 5), recovery with raptors/perchers (Fig 5), and possession of characters considered adaptive for arboreal locomotion (e.g. small size, pennaceous feathers, hindlimb feathering, elongate forelimbs, swivel wrist joints) [24,82,83].

One *Microraptor* claw was classed as perching whilst another from the same specimen was classed as predatory. We hypothesize the sharply curved pedal claws of *Microraptor* may straddle the perching/predatory morphospace (Fig 4) as fossil evidence suggest they were likely adapted for performing both scansorial and predatory functions For example, an articulated enantiornithine bird has been found within the gut of one specimen, strongly suggesting the animal hunted in trees [84]. In addition, biomechanical models indicate gliding ability [75,85], and extensive hindlimb feathers would have likely hampered terrestrial locomotion [84]. However, fish remains have also been attributed as gut contents for one *Microraptor* specimen [86], which would suggest the animal was not an arboreal specialist. The results herein reflect this conflict as predictions conflicted and posterior probabilities were low (Table 5); further studies and fossil evidence are needed to resolve this controversy.

One dromaeosaur (*Halszkaraptor*) plots outside the ‘predatory’ morphospace despite having a carnivorous lifestyle, which supports our interpretation of the measured ‘predatory’ morphospace as, more specifically, ‘predatory-perching’. *Halszkaraptor* may represent an abnormal data point as it has been interpreted as a semi-aquatic animal [37], but as semi-aquatic birds clustered with terrestrial taxa (see S1 Table) we expect the halszkaraptorine claw should not possess unusual adaptations. *Halzskaraptor*, one anchiornithid *Eosinopteryx*, and the troodontid *Borogovia* received robust terrestrial classifications (Fig 4, Table 5) consistent with osteological features and findings of past studies [28,30,37], and so these predictions suggest phylogenetically high curvatures of paravian claws may not influence false confirmations of arboreality as has been previously suggested [87–89].

Interestingly, all claws belonging to *Confuciusornis* were classed as predatory by all models (Fig 4,Table 5). A perching lifestyle is strongly supported by claw and toe proportions as well as the presence of an opposable hallux [77]. Morphology and inferred softness of the beak are traditionally regarded as evidence of herbivory [90,91] and so the predatory classification is controversial. Some workers have suggested softness of the horny beak is a taphonomic artefact [92], and fish remains have, though somewhat equivocally, been labelled as gut contents for one specimen [93]. However, conclusive, direct evidence of diet is unknown for *Confuciusornis* specimens [92]. As some *Confuciusornis* claws fall outside of extant morphospaces (Fig 4), and a predatory ecology is not well-supported by the literature, more evidence is needed to infer predatory habits for *Confuciusornis*.

Predictions of predatory ecology for two *Jeholorni*s claws contradict direct evidence of granivory for at least five specimens preserving seeds in the gut [92]. One claw received a perching prediction and so it seems probable based on this, direct dietary evidence, and skeletal characters that *Jeholornis* was a non-predatory perching bird that the model struggled to classify. Claws from *Sapeornis* were classed as either perching or scansorial, either of which could be argued for the taxa based on skeletal characters [94]. Other early avians *Zhouornis*, *Eopengornis*, *Parapengornis*, and *Fortunguavis* received predictions of terrestrial and perching but generally could not be well-resolved. Early birds were well-diversified by the late Mesozoic [95], and so it is possible that terrestrial and perching lifestyles are represented in the dataset. However, *Zhouornis* received conflicting predictions for claws of the same specimen, and the perching prediction for *Fortunguavis* contradicts interpretations of a scansorial lifestyle based on skeletal characters [27].

Peters and Görgner [12] have suggested that claw curvature cannot be correlated with an arboreal lifestyle because cliff-dwelling birds also possess sharply curved claws to cling to rocks. It is true that this hinders classification for a fossil taxon like *Archaeopteryx*, for which habitat structure is contested [57,80,87]. However, for animals that lived in the forests of Jehol [96] (*Microraptor*, *Changyuraptor*, *Xiaotingia*, *Zhenyuanlong*, *Jeholornis*, *Sapeornis*, *Confuciusornis*, *Fortunguavis*, *Eopengornis*, *Parapengornis*) or Daohugou [97] (*Pedopenna)*, it seems reasonable to conclude that curved pedal claws, in conjunction with other features such as elongate forelimbs and flight ability, represent arboreal adaptations.

The models occasionally yielded conflicting predictions for left and right PD-III claws belonging to a single fossil specimen. This could be caused by natural variation within the population, taphonomic distortion, or some unknown factor. The measured fossil claws did not exhibit any obvious distortion and often plotted within the overlap between morphospaces, and so it is plausible slight, naturally-occurring differences between claw angles could result in conflicting predictions. Conflicting predictions also occurred between specimens for *Jeholornis*, *Sapeornis*, and *Archaeopteryx*. In addition to previously suggested causes, it is possible these conflicting predictions occurred because the specimens represent different species or even individuals with different modes of life. Regardless of causative factors, conflicting predictions hinder classification of fossil taxa and should be considered in any future work seeking to class fossil taxa based on claws.

## Conclusions

Analysing ungual bone morphology has clear benefits in palaeontological study. The study found that curvatures of the PD-III ungual bone not only provide a useful proxy for certain modes of life but in fact exhibit a stronger correlation with lifestyle (accuracy = 0.7865) than do similar measures taken on claw sheaths of the same digit (accuracy = 0.7273). However, utilising solely curvatures of PD-III ungual bones to predict lifestyle is ill-advised because morphospaces overlap, and it is difficult to determine if fossil and extant claws are truly comparable based on these results. We suggest curvatures of fossil ungual bones may be useful when studying fossil taxa that lack well-preserved claw sheaths as they exhibit a strong correlation with behaviour and ecology for modern birds and squamates, but other lines of evidence such as other skeletal modifications [39], palaeoenvironmental data, biomechanics, and other aspects of claw morphology should also be considered [71]. The use of a digital dental X-ray system for making skeletal measurements from extant material proved to be extremely effective in this case and we would recommend this approach be considered as part of an extending morphological toolkit.

## Supporting information

S1 Table Extant sample taxa. (XLSX)

S2 Table Fossil taxa. (XLSX)
